# Cardiolipin is required *in vivo* for the stability of bacterial translocon and optimal membrane protein translocation and insertion

**DOI:** 10.1038/s41598-020-63280-5

**Published:** 2020-04-14

**Authors:** Sergey Ryabichko, Vilena de Melo Ferreira, Heidi Vitrac, Ramziya Kiyamova, William Dowhan, Mikhail Bogdanov

**Affiliations:** 1 Department of Biochemistry and Molecular Biology McGovern Medical School at the University of Texas Health Science Center, Houston, Texas 77030 USA; 2 Lewis-Sigler Institute for Integrative Genomics, Princeton University, Princeton, NJ 08540 USA; 3 Monash University, the Monash Institute of Pharmaceutical Sciences 381 Royal Parade, Parkville, VIC 3052 Australia; 4 Department of Biochemistry, Biotechnology and Pharmacology, Kazan (Volga Region) Federal University, Institute of Fundamental Medicine and Biology, Kazan, 420008 Russian Federation

**Keywords:** Membrane proteins, Phospholipids

## Abstract

Translocation of preproteins across the *Escherichia coli* inner membrane requires anionic lipids by virtue of their negative head-group charge either *in vivo* or *in situ*. However, available results do not differentiate between the roles of monoanionic phosphatidylglycerol and dianionic cardiolipin (CL) in this essential membrane-related process. To define *in vivo* the molecular steps affected by the absence of CL in protein translocation and insertion, we analyzed translocon activity, SecYEG stability and its interaction with SecA in an *E. coli* mutant devoid of CL. Although no growth defects were observed, co- and post-translational translocation of α-helical proteins across inner membrane and the assembly of outer membrane β-barrel precursors were severely compromised in CL-lacking cells. Components of proton-motive force which could impair protein insertion into and translocation across the inner membrane, were unaffected. However, stability of the dimeric SecYEG complex and oligomerization properties of SecA were strongly compromised while the levels of individual SecYEG translocon components, SecA and insertase YidC were largely unaffected. These results demonstrate that CL is required *in vivo* for the stability of the bacterial translocon and its efficient function in co-translational insertion into and translocation across the inner membrane of *E. coli*.

## Introduction

Protein translocation across and into the cytoplasmic membrane of *Escherichia coli* is mediated by an essential multiprotein machinery, which transports and inserts the vast majority of proteins within the bacterial envelope. This machinery is comprised of two heterotrimeric complexes consisting of SecY, SecE and SecG forming a membrane-embedded protein-conducting channel SecYEG^1^ and SecD, SecF and YajC forming an accessory translocation complex SecDFYajC^2^. It is widely accepted that secretory proteins (periplasmic and outer membrane proteins) are targeted to the SecYEG translocon post-translationally by the ATPase SecA^3^. Alternatively SecA can associate with the ribosome through a ribosome-nascent chain (RNC) transient complex and therefore act co-translationally^4,5^. Thus, the co-translational mode of interaction of SecA with its secreted protein substrates^6^ and membrane-spanning proteins^7^ is not unprecendented and can contribute to and co-exists with the post-translational mode of targeting and translocation^4^. During post-translational targeting, secretory proteins are captured first by the cytoplasmic homotetrameric export-specific chaperone SecB, which keeps preproteins in a translocation competent partially unfolded state and prevents premature misfolding and degradation^8^. The SecB-preprotein complex is subsequently bound by SecA, a translocation ATPase, which also provides binding sites for preprotein mature domains^9^, anionic phospholipid^10^, SecYEG^11^ as well as direct driving force for preprotein translocation through ATP binding and hydrolysis^11,12^. SecG stimulates protein translocation by undergoing a membrane topology inversion cycle^13^ which is tightly coupled to its function and linked with the insertion-deinsertion cycle of SecA. Highly hydrophobic protein substrates are delivered to SecYEG cotranslationally via a pathway that requires their interaction with the prokaryotic signal recognition particle (Ffh) followed by formation of a RNC complex, which is targeted to the SecY-bound Ffh receptor FtsY^14,15^. Membrane protein integrase (YidC) is engaged with SecYEG^16^ via its transmembrane and periplasmic regions^17^ to comprise the holotranslocon SecYEGDF-YajC-YidC^18^.

YidC functions as an intramembrane chaperone and insertase interacting with released non-mature membrane proteins at amphiphilic protein–lipid interface and facilitating insertion of transmembrane domains into the lipid bilayer where membrane proteins adopt their functional conformation^19^. Although being fully active as a monomer, homodimeric YidC can bind two substrate molecules simultaneously with only one active protomer being sufficient for YidC activity^20^.

The oligomeric arrangement of functional SecYEG translocon in the membrane is still matter of debate. Single SecY molecules are sufficient for SecA-mediated protein translocation *in vitro*^21^ and *in vivo*^22^. However, no oligomerization was detected via fluorescence-correlation spectroscopy experiments upon interaction with preprotein and SecA. Translocation intermediates can be generated *in vivo* with just one SecY copy^21^. This view is supported by X-ray data^23,24^. Single-particle cryo-EM^25^ analysis demonstrated that the SecYEG complex bound to a 70S translating ribosome and reconstituted in a nanodisc adopts a single channel configuration. Nevertheless, defective SecY can be rescued for translocation by linking it covalently with a wild-type SecY copy^26^ suggesting that protein translocation can be mediated by the oligomeric state of the SecY complex with only one SecY copy forming the channel. Whether a second SecY molecule prevents dissociation of SecA from the translocating SecY copy, thereby enhancing the processivity of SecA during translocation of a polypeptide chain^26–28^, is still unknown. The interaction with the non-translocating copy could prevent complete detachment of SecA during the nucleotide hydrolysis cycle and thus ensure processivity during polypeptide translocation. At the same time dimeric SecYEG was shown to be able to trap arrested pre-proteins based on cross-linking studies, demonstrating that SecYEG could function as a dimer at the membrane^29^ and form a high affinity binding site for dimeric SecA^30^.

Translocation of preproteins across the inner membrane requires anionic lipids by virtue of their negative head-group charge^11^ either *in vivo*^31,32^ or *in situ*^33^. *In vitro* translocation of pro-OmpA, the precursor of outer membrane protein is severely impaired in the absence of phosphatidylglycerol (PG) and cardiolipin (CL)^32^. However, these experiments did not allow an evaluation of the separate roles of monoanionic PG and dianionic CL in translocaion process. Recent *in vitro* experiments with nanodiscs demonstrated that both PG and CL were equally potent^34^. However, CL co-purified with SecYEG was shown to be required for stability of the SecYEG dimer *in vitro*^35^. CL specifically drives SecYEG dimerization *in silico*^36^ and stimulates both SecA ATPase activity^35^ and SecYEG translocation activity *in vitro*^36^. The dimer-promoting effect of CL was originally assessed by using a nonspecific photo-crosslinking. It was concluded that *in vitro* CL influences the stability of the SecYEG dimer, which itself serves as a high-affinity binding platform for SecA^35^. The dimer stabilized by CL also forms a cross-link between two SecE subunits consistent with the “back-to-back” arrangement of SecYEG complexes with adjacent SecEs at the dimer interface^35^. Liposomes containing a single SecY molecule had a reduced translocation activity compared with vesicles containing dimeric SecY^27^. In nanodiscs, the dimer is predominantly arranged in a “back-to-back” manner^28,37^ to comprise an active translocon, supporting the idea that the “back-to-back” orientation might be thermodynamically stable. Lipid nanodiscs containing only one SecY molecule did not stimulate ATPase activity of SecA, in contrast to nanodiscs containing two SecY copies. Thus, the SecY dimer together with CL supports the activation of the SecA translocation ATPase.

SecA is thought to act as a molecular motor driving translocation of the preprotein across the membrane by repeated ATP-driven cycles of insertion and retraction at the translocon channel. ATP hydrolysis is coupled to translocation of precursors^3^. The ATPase activity of SecA with a bound protein precursor is maximally activated when it engages SecYEG and anionic phospholipids^32^. Loose coupling to ATP hydrolysis can be a result of precursor polypeptides sliding backwards during translocation. SecA exhibits an equilibrium between cytoplasmic membrane and the cytoplasm^38^ and between monomer and dimer most likely representing an inactive and active translocation states, respectively. Although several results favor the monomeric functional state of SecA^39^, SecA appears to be predominately a dimer under physiological conditions^30,40^ where the dimeric state can be favored and maintained upon ATP and pre-protein binding^41^ and disfavored upon ATP hydrolysis^42^.

The energy provided by SecA dependent hydrolysis of ATP is required for protein translocation of secreted proteins across the SecYEG channel^43^ and large extramembrane domains of polytopic membrane proteins^44^. ΔΨ can contribute directly to translocation and drive translocation of preprotein residues or extramembrane domains containing large numbers of negatively charged amino acids electrophoretically^45–47^. SecDF may couple proton-motive force (PMF) to the release of the translocated polypeptides from SecYEG translocon^2^.

Knowledge of the role of lipid-protein interactions with respect to the lipid type and structure and the activity of multi-protein components of SecYEG translocase/YidC insertase is central to the mechanism of protein insertion and translocation and structure-function relationships in biological membranes. Whether *in vivo* protein insertion into and translocation across the inner membrane (IM) requires oligomers of the SecY complex and whether this arrangement is stabilized by CL is an important because it determines translocon efficiency.

The effect of *in vivo* CL depletion on protein translocation and assembly and stability of SecYEG translocon has not been studied on a molecular level. Our study aims to evaluate the i*n vivo* effect of CL on co- and post-translational translocation across and insertion into the IM. Herein, we studied the insertion and assembly of representative *E. coli* inner membrane (IM) α-helical and outer membrane (OM) β-barrel proteins as a function of cellular CL levels. To test whether translocation and insertion efficiency of several model proteins depends *in vivo* on the presence of CL, we analyzed translocation and insertion of several model proteins with established mode of translocation and inserion, SecYEG stability and SecYEG interaction with SecA in intact *E*. coli mutants that lacked all three CL syntases (ClsABC)^48^. Although the triple Cls deletion contains no detectible CL and elevated levels of PG, it showed no growth defects on rich media and is not temperature sensitive for growth as shown for yeast mutants lacking CL^49^. However, both translocation across and insertion into the IM of α-helical proteins and assembly of outer membrane β-barrel precursors were severely compromized in CL-depleted cells mainly due to destabilization of SecYEG dimeric complex. The absence of CL leads to inhibition of secretion alkaline phosphatase which is translocated mainly in post-translational manner. The biogensis of co-translationally inserted of lactose permease (LacY) was also impaired by the depletion of CL. The OmpF monomeric precursor accumulates as a translocation intermediate in the cell envelope in the absence of a CL. These effects are not due to a reduced membrane potential, which in turn can impair protein translocation into and across the inner membrane. Our results indicate that the lack of CL affects the organization and stability of the SecYEG/SecA translocon and is required for optimal translocation across and into the IM of *E. coli*.

## Results

### Experimental system

**Figure 1:**
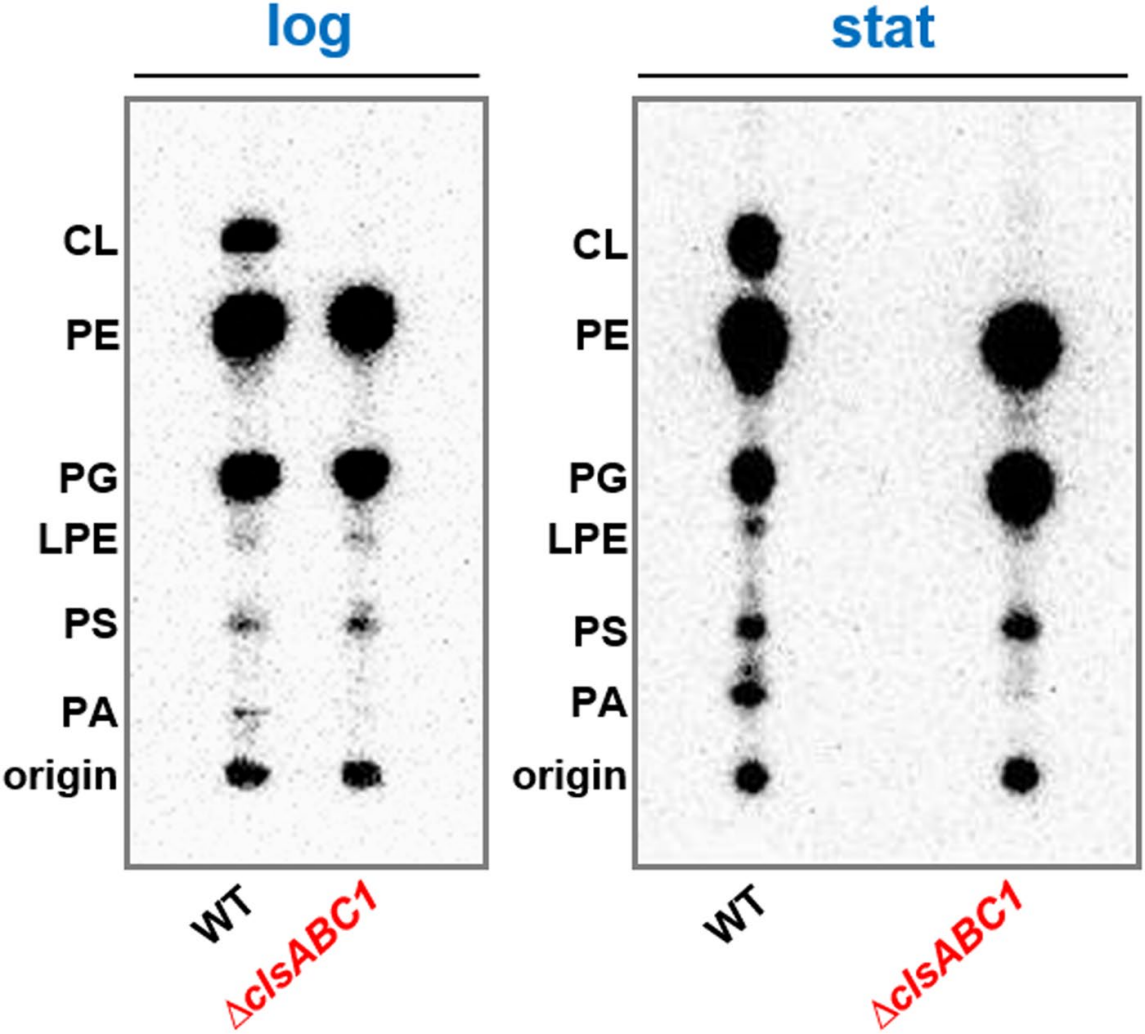
The ∆clsABC1 *E. coli* BKT12 mutant lacks detectible CL regardless of growth phase. The cells were grown in the presence of [^32^P]PO_4_. Strain W3110 (WT) and BKT12 ∆*clsABC1* strains with the indicated *cls* gene deletions were grown in LB medium containing 10 g/L of NaCl and [^32^P]PO_4_ to either mid-log phase (log) or to stationary phase (stat). Phospholipids were extracted and analyzed by one-dimensional TLC as described in Materials and Methods.

To define the molecular steps affected by absence of CL in protein translocation and insertion, we utilized *E. coli* mutant BKT12 that lacked all three CL synthases (ClsABC1)^48^. Although YmdB (C2) is required for full activity of the ClsC1-YmdB, complex, lack of ClsC1 prevents the utilization of PG and PE in a novel mode of CL biosynthesis^48^. [^32^P]PO_4_-labeling in conjunction with thin layer chromatography (TLC) demonstrates that the *∆clsABC1* mutant is devoid of CL in logarithmic and stationary phase grown cells, producing only PG as the major anionic phospholipid (Fig. 1). Therefore, this mutant can be utilized to separate the role of the two major anionic phospholipids, PG and CL in different cell functions.

### CL-depleted *E. coli* cells are impaired in preprotein translocation across the IM

**Figure 2:**
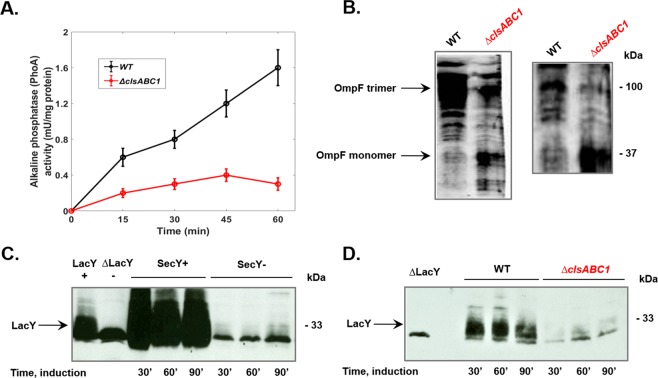
CL-lacking *E. coli* cells are severely impaired in protein insertion into and translocation across the IM. (**A**) Protein translocation of alkaline phosphatase is reduced in the absence of CL. The alkaline phosphatase enzymatic assay was used to monitor translocation across the IM of wild type (WT) W3110 (black line and CL-lacking BKT12 Δ*clsABC1* cells (red line). The efficiency of secretion of alkaline phosphatase was estimated by the indirect measurement of enzymatic activity in the periplasm as it is described in Materials and Methods. (**B**) The trimeric assembly pathway of OM β-barrel precursor OmpF is impaired in CL-lacking cells. Western blot analysis of total membrane fraction was utilized to assay co-translational translocation and maturation of this trimeric porin within the cell envelope with two different monomer-specific anti *E.coli* OmpF monoclonal antibodies. (**C**) Insertion yield of LacY was assayed in cold-sensitive *secY39*^*ts*^ mutant, which is well known for the rapid temperature response *in vivo* and impaired translocation activity at low temperatures^76^ to support the contention that LacY is inserted into IM a SecY dependent manner. The ∆LacY lane shows a non-LacY band that crossreacts with LacY-specific antibody. (**D**) Insertion yield of LacY was determined after induction of its synthesis in WT W3110 and BKT12 *ΔclsABC1*. Native copy of *lacY* gene was carried on plasmid pT7-5 (Amp^R^) and expressed under OP_tac_ regulation as previously described^74^. Immunoreacting LacY bands were visualized by ECL and quantified by BioRad imager and software. Broadening of LacY and OmpF protein bands is due to incomplete denaturation of membrane proteins and presence of multiple folding intermediates.

Although most bacterial secretory proteins destined beyond the IM are secreted post-translationally by the SecYEG translocon^50^, *E. coli* alkaline phosphatase and OmpA are widely used model proteins, which are capable for both co- and post-translational modes *in vivo* and *in vitro*^51,52^. Measurement of alkaline phosphatase enzymatic activity in intact toluene permeabilized cells is a reliable assay of translocation of preproteins across IM and enzymatic activity is used very often to quantitate the extent of translocon-assisted translocation *in vivo*^53^. Activity of this protein directly reflects translocation across the IM since alkaline phosphatase becomes active only after dimerization with the formation of two intramolecular disulfide bonds within each monomer in the periplasm^54^. The kinetics of appearance of alkaline phosphatase in the periplasm and steady state levels were strongly reduced in cells lacking CL (Fig. 2A).

### Biogenesis of β-barrel proteins is impaired in CL-depleted cells

Nascent substrate proteins including OmpF may interact with SecA long before they interact with SecB or productively engage with SecYEG. Irrespective of the binding mode of SecA either to ribosomes or the SecYEG translocon, OmpF translocation is frequently described as co-translational^4^. The translocation of this β-barrel precursor allows the analysis of distinct assembly steps, which can be visualized by semi-native gel electrophoresis and subsequent Western blotting with approproiate antibodies. Since proper assembly is required to expose the specific antigenic sites at the surface of the trimeric form of OmpF, detected immunorecognized conformations of trimers could be used as an index of productive assembly of porins^55^. Thus we decided to study the assembly of the trimeric porin OmpF by monitoring formation of the mature trimeric complex. The biogenesis of this assembled protein was impaired by the depletion of CL, since the OmpF monomeric precursor accumulates as a translocation intermediate in the cell envelope in the absence of a CL (Fig. 2B).

### Insertion of α-helical membrane protein LacY is impaired in CL-lacking cells

We also analyzed the biogenesis of lactose permease (LacY) that contains α-helical transmembrane segments by testing its yield of insertion into the IM. We confirmed that LacY is inserted into the IM in a SecY dependent manner (Fig. 2C) since the yield of insertion was severely inhibited in cold-sensitive secY39^ts^ mutant grown at a non-permissive temperature. The relative amount of LacY in exponentially grown cells was determined by quantitative Western blotting, using serial dilutions of the respective purified proteins as standards. We found that in CL-lacking cells LacY insertion yield is approximately one-tenth the level in wild type cells, comparable to the insertion of LacY into the IM of the cold-grown *secY39*^ts^ mutant (Fig. 2C,D).

### CL-lacking *E. coli* cells are capable of sustaining a wild-type proton electrochemical gradient

Protein translocation into and across the IM requires *in vivo* and *in vitro* the presence of both components (ΔΨ and ΔpH) of proton electrochemical gradient (Δμ_H_^+^)^56,57^ raising the possibility that the translocation and insertion defects observed in CL depleted *E. coli* may be related to a reduction of Δμ_H_^+^ in the absence of CL. In order to rule out a reduction in the components of Δμ_H_^+^ or membrane leakiness to protons as the basis for the reduction of protein translocation in the absence of CL, the ability of cells lacking CL to maintain a normal electrical potential across the membrane was experimentally determined. Wild type and BKT2 cells were employed to estimate ΔΨ and ΔpH using radioactive lipophilic cation triphenylmethylphosphonium (TPP^+^) and weak acid DMO distribution between the internal volume of intact cells and the cell medium as described in Methods. At an external pH of 7.5 all transport systems including SecYEG translocon should be driven by the electric component of Δμ_H_^+^. Since some transport systems are driven by the ΔpH component of the Δμ_H_^+^ at an external pH of 5.5, ΔpH was measured at different external pH values. We demonstrated that CL-lacking and WT celss displayed essentially the same bioenergetic parameters at pHs 5.5 and 7.0 (Table 1).

**Table 1:** Wild type and CL-lacking cells are able to maintain a WT level Δμ_H_^+^ at two different pH values of the growth medium.

		W3110	BKT12
**ΔΨ**	**pH 5.5**	−113 ± 8	−117 ± 6
	**pH 7.0**	−98 ± 6	−95 ± 6
**ΔpH**	**pH 5.5**	−75 ± 7	−70 ± 9
	**pH 7.0**	−35 ± 6	−38 ± 8

ΔΨ (mV) and ΔpH (mV) were determined from radioactive lipophilic cation TPP^+^ and weak acid DMO distribution between the internal volume of intact cells and the cell medium by the silicone oil-spin method. Values are means (± S.D.) from three experiments, where each determination was done in duplicate.

These data are consistent with recent finding demonstrating that values of membrane potential (measured with the fluorescent probe 3,3-dipropylthiadicarbocyanine iodide) were essentially the same in wild-type and BKT12 mutant cells grown in M9 glucose containing minimal medium^58^.

### The amount of SecYEG translocon and insertase (YidC) components and translocation accessory proteins (SecA) was not affected by the absence of CL

**Figure 3:**
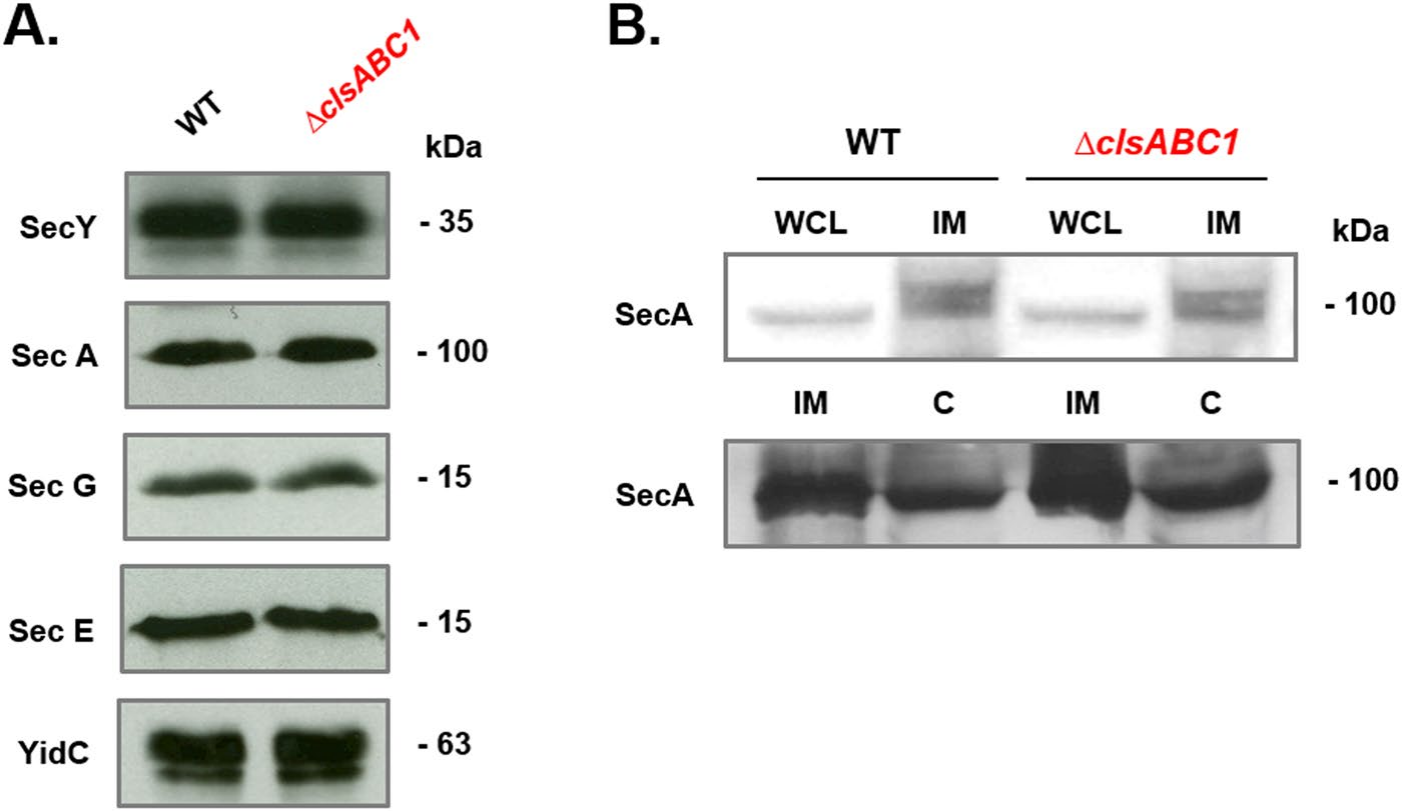
Abundance of core translocon components (**A**) and SecA (**B**) in CL-lacking *E. coli* cells. IM fractions **(A)** or whole cell lysates (WCL), IM and cytoplasmic fractions (**C**) **(B)** from WT *E. coli* W3110 and mutant BKT12 Δ*clsABC* were separated by SDS-PAGE and transferred to nitrocellulose membranes. Proteins were immunostained with antibodies against SecY, SecE, SecG, SecA, YidC. For each lane 10 μg of IM protein was loaded.

Alterations in the steady-state protein levels of translocon components and their stoichiometry could affect the kinetics and yield of translocation into the periplasm and insertion into the IM and OM. To address whether lack of CL may impair the above processes, we analyzed the abundance of SecYEG core translocon components. Interestingly, the amounts of SecYEG core translocon components, SecA and YidC were comparable in the CL-lacking mutant and WT cells (Fig. 3).

### CL is required *in vivo* for the maintenance of dimeric state of SecYEG

**Figure 4:**
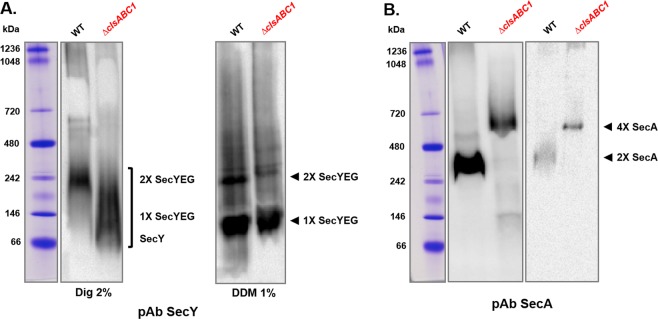
The translocon in WT *E. coli* membranes is organized as a SecYEG dimer, which is unstable in CL-lacking cells due to lack of this lipid or dissociation of SecA. The BN-PAGE pattern of the SecYEG **(A)** and SecA complexes **(B)** are altered in CL-deficient *E. coli* cells, indicating that CL is involved in the stability or organization of the translocase complexes. The IMs from wild-type *E. coli* W3110 and mutant BKT12 *ΔclsABC* were solubilized with 2% digitonin (1 μg digitonin/μg protein) or 1% DDM, separated by BN-PAGE, transferred to nitrocellulose membranes and immunostained with antibodies against SecY, SecE and SecG (the core subunits of the SecYEG translocon, **A**) and SecA (a peripheral component of translocon, **B**). For each lane 25 μg of IM protein was loaded. Immune staining was carried out with all 3 antibodies individually. All three antibodies recognized a single complex running at about 230 kDa, but only the Western blot of BN-PAGE-separated proteins immunostained with anti-SecY is shown. **(B)** Immunoreacting SecA assemblies were visualized at different loading concentations.

We next asked whether the alteration of the rate of alkaline phosphatase translocation, yield of LacY insertion and OmpF assembly is affected by the stability of the SecYEG complex. In order to characterize the stability and organization of SecYEG translocon, we employed BN (blue native)-PAGE, a technique widely used to analyze membrane protein-protein and protein-lipid interactions, the oligomerization properties and subunit composition of membrane protein complexes^59^. Pure IM vesicles containing only endogenous chromosome-borne amounts of SecYEG and SecA, were solubilized with 2% digitonin and subjected to BN-PAGE and Western blot analyses (Fig. 4A,B). The native protein patterns of SecYEG were altered in the absence of CL confirming that the stability of SecYEG was considerably decreased in CL-depleted cells. In WT extracts the SecYEG translocon forms dimeric complexes migrating as a discreet 242 kDa species, which is dissociated and shifted in CL-lacking cells to faster migrating diffuse species (Fig. 4A) composed from monomeric SecYEG and SecY in the accordance with their molecular weight (~140 and ~66 kDa, respectively)^30^. Only SecYEG monomers were visualized in 1% DDM (Fig. 4A, right panel) supporting previous results^29^ demonstrating that the SecYEG monomer/dimer ratio depends strictly on the DDM concentration. These results mirror the use of digitonin in solubilization of bacterial translocons and many other supercomplexes including mitochondrial respirasomes. The yeast respirasome composed of complexes III and IV displays as a supercomplex on BN-PAGE when exposed to only digitonin but dissociates to its individual complexes in the presence of DDM^59^. Addition of CL, and no other phospholipid, reconstitutes the supercomplex^60^.

Therefore, these mobility shifts may indicate that CL is bound to SecYEG in wild-type cells and critical for the stability of the dimeric translocon. In previous studies using BN-PAGE, multiple SecYEG complexes have been detected in IMs isolated from SecYEG over-expressing strains and solubilized at different protein/detergent ratio^29,30^. We focused on the SecYEG complex at a defined 1:1 protein/digitonin ratio and detected predominantly the 220–240 kDa complex corresponding to the SecYEG dimer in WT cells. Our data are consistent with other results^61–63^ and in full agreement with the mobility of purified SecYEG translocon from the IM of wild type *E. coli*^30,64^ demonstrating that translocon is organized in wild type cells as SecYEG dimer as detected with antibodies directed against SecY, SecE, or SecG^62^. The observed mobility difference from the calculated molecular weight of 150 kDa for the SecYEG dimer and 75 kDa for the monomeric SecYEG complex most likely reflects binding of detergent and Coomassie Blue to the SecYEG complex^59,62^.

### Whether CL is required for the stability of the SecYEG /SecA complex?

As was demonstrated *in vitro*, CL stabilizes the SecYEG dimer for its function as an high affinity binding platform for SecA^35^. However SecA itself is primed allosterically for high affinity binding to SecYEG via interaction of its positively charged N-terminus with negatively charged lipids^10,65^. Thus alternatively, lack of CL could also result in dissociation of a non-translocationally efficient conformer of SecA from SecYEG along with destabilization of the SecYEG dimer. We asked whether CL is required for not only stability of the SecYEG dimeric complex but also for its interaction with SecA. In the absence of CL or presence of unstable SecYEG dimer SecA may display altered interfaces and configurations that could lead to formation of different oligomeric assemblies^30^. Indeed the relative migration of native SecA assemblies was drastically altered in the cells lacking CL (Fig. 4B). SecA solubilized from IM of the WT or BKT12 strain migrated as a ~270 kDa dimer or a tetramer running above 480 kDa, respectively. Thus relative proportions of these two oligomeric forms could depend on their interaction with CL and SecYEG. High likelihood of SecA dimers is already predetermined by its the dissociation constant, however, the presence of different binding partners (anionic lipids, SecYEG, nucleotides) can directly affect the oligomeric state^40,66,67^. Due to transient and unstable interactions between nascent SecA and SecYEG the *bona fide* SecA-SecYEG associations are not detected usually by BN-PAGE unless two protomers are crosslinked to form a dimer^30^, which forms a stable binding site for SecA monomers and dimers. A second SecY molecule may prevents dissociation of SecA from the translocating SecY copy, thereby enhancing the processivity of SecA during translocation of a polypeptide chain^26–28^. Crudely extracted SecA usually exhibits a complex behavior equilibrating between physiologically relevant monomeric and dimeric forms and also tetrameric forms as was shown for their purified counterparts^30^. In CL-lacking IM the translocationally inefficient conformer of SecA dissociated from monoanionic PG and/or losely bound unstable SecYEG dimer aggregates into a tetrameric forms suggesting that oligomerization properties of SecA are affected by absence of CL.

## Discussion

Here, we tested the *in vivo* role of CL in stabilization of the dimeric state of the protein-conducting channel SecYEG^35,36^ and its function as the bacterial translocon responsible for protein translocation and insertion of periplasmic, IM and OM proteins. Previous postulation for a requirement for efficient function of the protein translocation machinery relied on *in vitro* and *in silico* data. In the absence of CL we show that periplasmic alkaline phosphatase, OM protein OmpF and IM protein LacY are translocated, assembled or inserted into the IM, respectively, less efficiently (Fig. 2). Our results strongly suggest that this deficiency is due to a less stable SecYEG dimeric complex and weaker or altered mode of association of SecA with either the membrane or SecYEG (Fig. 4). Translocation and insertion defects are not due a reduced membrane potential (Table 1), the amount of core translocon subunits or YidC (Fig. 3), which would impair protein translocation into and across the IM. Recently, it was demonstrated that a reduction in CL levels impairs translocation across the IM of alkaline phosphatase expressed under a regulated promoter and monomeric OM OmpA protein of *E. coli* cells^53^ grown under nutrient limited condition. CL-containing cells produce mature OmpA at a much faster rate than CL-depleted *ΔclsABC1* cells^53^. The authors suggested that these bulk secretion and folding defects in turn can somehow activate the Rcs pathway via the OM lipoprotein RcsF. Accumulation of the slowly migrating unfolded form of OmpF employing a “heat-modifiability” gel-shift assay, which distinguishes folded and unfolded populations of OmpF, indicated also maturation and folding defects of β-barrel proteins assembled in cells lacking CL^58^.

Although visualization of SecYEG ensembles in *E. coli* membranes depends on the expression level and the solubilization conditions^29,30,41,61^, there now seems to be consensus that the SecYEG channel predominantly exists as a dimer^62,64,68^, which is further supported by our data. Our analyses using *E. coli* IM with normal chromosome borne levels of SecYEG and SecA reveal very distinct altered BN-PAGE patterns for the SecYEG and SecA complexes without CL. When CL is present the SecYEG translocase forms dimeric complexes, which are dissociated and shifted to faster migrating forms in CL-lacking cells. This mobility shift may indicate that CL is bound to SecYEG in WT cells and is critical for the stability of the translocase. In the absence of CL the SecYEG complexes migrated on BN-PAGE within a range of ~70–240 kDa suggesting that SecYEG translocon organized as a dimer in WT cells is unstable in IMs lacking CL (Fig. 4A).

**Figure 5:**
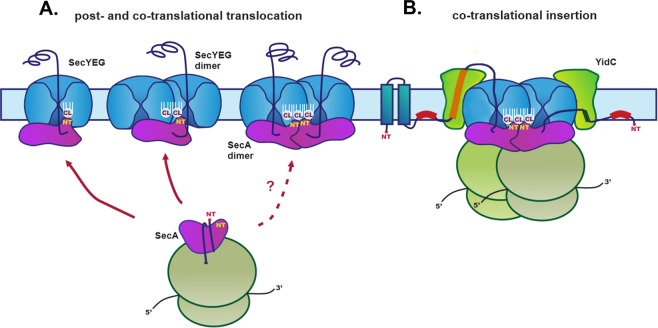
Translocon and CL mediated protein co- and post-translational membrane translocation and co-translational insertion of proteins in *E. coli*. **(A)** Proteins are delivered to the SecYEG translocon by SecA-dependent pathway utilized by secreted periplasmic and OM proteins. Although a single copy of SecY is sufficient for simultaneous binding to SecA and translocating a substrate (left), a second SecYEG molecule maybe required for efficient SecA binding^26^. A scenario in which SecA would still bind to one copy of SecYEG but translocation would proceed through the a second SecYEG is shown (second from left). The functional oligomeric state of SecA during the protein translocation cycle is still a controversial issue. Both monomeric and dimeric forms have been suggested to be physiologically relevant. The SecA monomers (or dimers, dashed line) engage transiently with the SecYEG. The SecA monomers can co-translationally recognizes the nascent chain of an IM protein with high affinity and specificity^7^. **(B)** During lateral discharge from the dimerized SecYEG channel, a nascent IM protein interacts with YidC, an intramembrane insertase which assists in sidewise release of membrane proteins. The stability of the dimeric SecYEG translocon and its association with SecA is altered in CL-depleted cells (Fig. 4) indicating that the lack of CL destabilizes these interactions, thus affecting all possible scenarios shown in the diagram. Efficient binding of SecA to a translocating SecY channel requires that SecA first interacts through its N-terminus with negatively charged lipids in the membrane^10^, which enriches SecA in the vicinity of SecYEG. SecA drives translocation through the SecYEG channel by ATP hydrolysis. When SecA is lost from SecY in its ADP-bound state, the polypeptide chain is no longer pushed into the channel^11^. In the presence of dianionic CL, the affinity of SecA for the membrane and/or SecYEG is stronger and SecA is more frequently associates with the SecY channel, thereby indirectly accelerating the translocation process. Thus, CL in the vicinity of SecYEG dimer can contribute to appropriate timing and efficiency of SecA cycling between high- and low-affinity states determined by the equilibrium between ADP- and ATP bound states.

We conclude that CL is required for the efficient function of the translocon and depletion of CL destabilizes the dimeric state of SecYEG and disfavors its association with SecA or *vice versa*. However, we cannot exclude that these faster moving SecYEG complexes represent actively translocating machines, which are still organized as short-lived dimers in CL-depleted cells. This interpetation is in agreement with recent results suggesting the contacts between the two SecYEG protomers are destabilized during the translocation process and an actively translocating SecYEG dimers are not stable enough to be visualized by BN-PAGE^64^. Regardless of which scenario, our observations reflect the central role of the intimate interplay between membrane-embedded protein translocon and the surrounding phospholipid environment (Fig. 5).

A second SecY molecule can prevent the dissociation of SecA from the translocating SecY copy, thereby enhancing the processivity of SecA during translocation of a polypeptide chain^26–28^. However, it is still unclear whether SecA binds directly to SecYEG or this binding is mediated by anionic lipids. SecA can bind directly to the SecY complex in detergent, but it can also interact with negatively charged lipids^65^. Even in the absence of SecYEG, SecA binds to liposomes that contain anionic lipids. Deletion of the N-terminus results in the inactivation of SecA, but activity can be restored by replacing the N-terminus with a His-tag and supplementing SecYEG proteoliposomes with Ni-NTA (nitrilotriacetic acid) lipids, suggesting that tethering to a negatively charged membrane is important for translocon functioning^11^. It was demonstrated that efficient rebinding of SecA to a translocating SecY channel^11^ requires that SecA interacts first through its N-terminus with negatively charged lipids in the membrane^10^. Indeed acidic phospholipids allosterically activate SecA for ATP-dependent protein translocation^69^. These events can either intiate a local interface enrichment or restrict diffusion of SecA in vicinity of SecYEG translocon^11^ or lead to large conformational changes in SecA^10^ thus either indirectly or directly increasing the rate of association of SecA to the protein conducting channel. In the presence of dianionic CL, the affinity of SecA for membrane and/or SecYEG could be stronger and SecA could be more frequently associate with SecYEG channel. Without CL SecA would be more frequently dissociated from the translocating SecY channel and therefore retard the translocation process. Highly dynamic and cycling associations and disassociation interactions of SecA with SecYEG translocon during translocation process (often referred as “SecA processivity”) can be obligatory for efficient translocation of given substrate^70^. Although SecA is associated with SecYEG pre-embedded into nanodiscs regardless of the anionic lipid type present, SecA binding affinity with CL was more than twice as good as with PG as followed from corresponding K_d_s determined by Microscale Thermophoresis^71^. These data are consistent with *in silico* occupancy analyses, which clearly demonstrated that even both PG and CL are able to provide the same stabilizing interactions in the proximity of SecY lateral gate but CL enhances stabilizing effect through a tighter binding to both N-terminus and nucleotide bindingat site 2 of SecA as followed from lipid relative residence times.

SecYEG is able to interact with MPIase, a novel bacterial membrane *Escherichia coli* glycolipid (comprising a glycan chain with three N-acetylated amino sugars connected to diacylglycerol through a pyrophosphate linker), which is required for *in vitro* transmembrane SecG dynamics and allows two SecYEG monomers to adopt “side-by-side” orientation, an arrangement which appears to be important for overall (pre)protein insertion and translocation process^72^. On the other hand, it was shown that dimer stabilized by CL forms a cross-link between two SecE subunits consistent with the “back-to-back” orientation of SecYEG complexes, in which both protomers interact via a SecE-SecE interface^35^. In nanodiscs, the dimer is predominantly arranged in a “back-to-back” manner^28^ to comprise translocon in thermodynamically stable conformation. If two interaction surfaces in the SecY complex can be used at the same time^22^, interplay between CL and MPIase could be necessary for retention of either “back-to-back” or “side-by-side” dimeric SecYEG structure or shift from equilibrium if both orientations can exist transiently in the resting state of translocon. If dynamically and reciprocally driven CL and MPIase driven SecYEG monomer reorientations exist during catalytic cycle of translocation, it would imply that not only the monomer–dimer transition but even the transition between differently oriented dimers can take place during translocation acts as was proposed^72^.

Recently two high and mid-occupancies CL binding sites within transmembrane domains (TMDs) 2–3 (site 1) and TMD1 and TMD2 of SecY respectively were predicted by Coarse-grained Molecular Dynamics simulations and validated further by site-directed mutagenesis and Native Mass Spectrometry, which demonstrated significant decrease in CL binding to mutant SecY molecules with elimination of positively charged amino acids at these sites^36^. Presence of CL molecules at these two sites appears to be required for of the maintenance of high ATPase activity of SecYEG associated SecA and stimulation of protein translocation by PMF, since inside out vesicles (ISOv) made from WT type *E. coli* CL-containing cells expressing site 1 and site 2 mutants or ISOv made from triple *clsABC* mutant cells but expressing WT type SecYEG lose this ability^36^. Although *in vivo* CL-depleted cells are able to maintain both components of proton motive force (Table 1) and PG^71^ and CL^36^ transient binding sites overlap at the interface between SecG and SecY, SecYEG/SecA can function *in vivo* in the fully or partially uncoupled translocation non-efficient mode due to presence of PG instead CL at these distinct regions. Thus CL could be involved *in vivo* directly in molecular mechanism for two modes of energy coupling in SecA/SecYEG ATP/PMF driven protein transport.

Although *in silico* coarse-grained dimer analysis demonstrate that identified CL specific binding sites are not involved in SecYEG dimerization events, CL is still driving formation of SecYEG dimers within a given simulation time frame^36^ Our *in vivo* results are consistent with this study. Thus CL molecules can provide direct or indirect structural constraints for SecA/SecYEG translocon stabilization and efficient function in living cells.

We don’t know whether dissociation of SecA caused by distortion of its interactions with monomerized SecYEG or loss of SecA direct interactions with anionic lipids can occur prior its engagement with SecYEG and unable to yield highly active translocons. However, in the light of the chemical, structural, and physical complexity of energized bacterial biological membranes and the presence of transmembrane electrochemical gradients and electrostatic potentials, the function of SecYEG and translocation of nascent preproteins could be subjected to different lipid-specific regulatory mechanisms that are only now beginning to be uncovered.

## Materials and Methods

### Reagents

[^14^C] 5,5 dimethyloxazolidine-2,4-dione-2-^14^C (DMO) was generous gifts of Dr. H. R. Kaback (University of California, Los Angeles). Tetraphenylphosphonium [^3^H]-TPP came from Amersham. SuperSignal West Pico chemiluminescent substrates for detection of horseradish peroxidase came from Thermo Fisher Scientific). Antibodies directed against core translocon components, YidC and OmpF were a generous gift of Professors Ken‐ichi Nishiyama (Iwate University, Morioka, Japan), Tassos Economou (KU Leuven - University of Leuven) and Ross Dalbey (Ohio State University), George Koch (Institute of Biochemistry and Molecular Biology, Albert-Ludwigs University Freiburg) and Hiroshi Nikaido (University of California, Berkeley), respectively. Molecular biology grade chemicals were purchased from Sigma.

### Bacterial strains and growth conditions

*E. coli* W3110 is designated as wild type (WT) with respect to its glycerophospholipid composition. To investigate the effect of deletion of all three predicted *cls* genes a previously constructed BKT12 (*∆clsA, ∆clsB, ∆clsC1*::Kan^R^) was utilized^48^. Liquid LB media was made with 10 g of tryptone, 5 g of yeast extract, and 10 g of NaCl per liter (high salt LB Miller medium) and was the normal media for growth in all experiments utilizing [^32^P]PO_4_ labeling_,_ enzyme and energetic parameters assays, membrane preparation. Strains were routinely grown at 37°C and cell density was measured as absorption at 600 nm (A_600_). Cold-sensitive *E. coli* mutant AD208 (MC4100 *secY39*^*ts*^ *zhd-33::Tn10*) and and parent strain AD202 (MC4100 *ompT::kan, SecY*^+^) was a generous gift of Professor Koreaki Ito (Kyoto University). SecY39^ts^ mutant cells were growing first at 37 °C until logarithmic stage of growth and shifted then to growth at non-permissive temperature (23 °C).

### Lipid extraction and TLC analysis

To determine the steady state phospholipid composition by radiolabeling, cells were uniformly labeled with 5 µCi/ml of [^32^P]PO_4_ after dilution to A_600_ of 0.05 to initiate logarithmic growth or stationary growth after dilution of the overnight culture. Cells were harvested by centrifugation and phospholipids were extracted by acidic Bligh Dyer procedure and analyzed after separation either by one-dimensional TLC on boric acid impregnated silica gel plates (chloroform/methanol/water/ammonium hydroxide (60:37.5:3:1, v/v). For TLC, 20 X 20 cm high-performance Partisil LK5 silica gel precoated TLC plates with a concentration-zone (Whatman Inc. Clifton, NJ) were impregnated for 1 min in 1.2% boric acid in ethanol-water (1:1) as described^48,73^. Radiolabeled lipids were visualized and quantified using a Personal Molecular Imager^TM^ FX (Bio-Rad Laboratories). Stored images were processed and quantified using Quantity One software for scanning and analysis of the captured Phosphor images (Bio-Rad Laboratories). Phospholipid content is expressed as mol% of total phospholipid (correcting for two phosphates per molecule of CL) based on the intensity of the captured signal on Phosphor screen generated by the radiolabeled spots on the TLC plate. The results presented are representative of two or more determinations.

### ΔΨ and ΔpH determination

An electrical potential and pH difference across the membrane (ΔΨ and ΔpH, respectively) were examinated by partitioning of radiolabeled radiolabeled probes using the silicone oil-spin method^48,73^. Cells (200 ml) were harvested during mid-log phase of growth by centrifugation and resuspended (5 ml) either in the 100 mM HEPES /KOH buffer (pH 7.5) or 100 mM MES/*K*OH buffer (pH 5.5) to an A_600_ of 10–15 (10–15 mg of cell protein/ml), respectively.

The difference in pH across the membrane of the *E. coli* cells was measured by determining the difference in the amount of radiolabeled DMO associated with cells before (energized cells) and after (de-energized cells) treatment with 1.7% toluene or heating at 60 °C for 30 min. This approach provides a correction for nonspecific binding of DMO. At 25 °C steady-state distribution of DMO was obtained within 2 min and remained constant for at least 10 min. After 3 min of incubation, 0.5-ml samples were removed from the reaction mixtures (cells suspended in 100 mM MES/KOH (pH 5.5), 150 mM KCl, 16 mM succinate, [^14^C]DMO (0.25 μCi/ml, 60 μM final concentration) and layered onto 0.15 ml of 22% perchloric acid and 0.50 ml of silicone oil (d = 1.05, Aldrich) in microcentrifuge tubes, and centrifugated through the silicone oil in an Eppendorf microcentrifuge at 20,800 × g for 5 min at room temperature; a de-energized sample was treated in the same way. After centrifugation, 0.05-ml samples of the perchloric acid phase and the supernatant remaining above the silicon oil were removed for scintillation counting after drying of aliquots on GF/F Whatman glass microfiber filters. The internal pH (pH_in_) was calculated from the Nernst equation as follows (1):

1 $${{\rm{pH}}}_{{\rm{in}}}=p{K}_{a}+\,\log \left\{\,\frac{{[{\rm{DMO}}]}_{{\rm{in}}}-{[{\rm{DMO}}]}_{{\rm{nonspecific}}}}{[{\rm{DMO}}]{\rm{out}}}\cdot [{10}^{{{\rm{pH}}}_{{\rm{out}}}-p{K}_{a}}+1]-\,1\right\}$$

where p*K*_a_ is the negative logarithm of the apparent ionization constant of DMO (approximately 6.32 at 25°C), pH_out_ is the extracellular pH, DMO_in_ is the intracellular conc entration of DMO, DMO_out_ is the extracellular concentration of DMO. The ΔpH is the difference betw een pH_in_ and pH_out_ (alkaline inside). At pHout > 7.4, pH_in_ is more acid than pH_out_. The intracellular water was determined from measurements of distribution of non-permeant non-permeant hydroxy[^14^C]methyl-inulin and ^3^H_2_O and was taken as 6.34 μl/mg of cell protein of *E. coli* cells^74^.

An electrical potential across the membrane ΔΨ was determined by tetraphenylphosphonium ^3^H-TPP technique and silicone oil-spin method^73^. To measure ΔΨ the cells (5 ml from above) were preincubated in 100 mM tris-HCl buffer pH 8.0 with 0.5 mM of EDTA (from 10 mM pH 7.5) at 37 °C for 2 min. The treatment was stopped with 10X of ice cold 100 mM HEPES /KOH buffer (pH 7.5). The cells collected at 20,800 × g for 5 min and washed once with ice cold 100 mM HEPES /KOH buffer (pH 7.5) and resuspended in original volume (5 ml) in the same buffer. The above method was also used to verify that 50 μM FCCP fully de-energized cells at extracellular pH 5.5 and 150 mM potassium ion. Energized and de-energized cells cells were equilibrated with the tetraphenylphosphonium cation (10 μM, 0.5 μCi/ml) for 5 min and subjected to silicone oil-spin method.

The ΔΨ was calculated by substitution of concentration ratios into the Nernst equation as follows (2):

2 $$\varDelta \psi =-61\,{\rm{lg}}\,\frac{{[{{\rm{TPP}}}^{+}]}_{{\rm{in}}}-{[{{\rm{TPP}}}^{+}]}_{{\rm{nonspecific}}}}{{[{{\rm{TPP}}}^{+}]}_{{\rm{out}}}}$$

### Whole cell lysate preparation

Cells were washed from growing media by 20 mM Tris-HCl, pH 7.5 and homogenized on ice in lysis buffer (20 mM Tris-HCl, 1% Triton X-100, 100 mM NaCl, 1mM EDTA, 1 mM PMSF, pH 7.5). Afterwards they underwent ultrasound sonication and were incubated on ice for 30 min. The lysates were centrifuged at 8,000 × g (Sorvall RC6 centrifuge, SS-34 rotor) for 10 min and supernatant was used as a whole cell lysate.

### Isolation of IM by differential centrifugation

The cells were resuspended in 0.1 M Tris-HCl, 10 mM MgCl_2_, 1 mM PMSF, pH 8.0. After passing through French press (8,000 psi), unbroken cells were removed by centrifugation at 30,000 g (Beckman SW-41 rotor) 4 °C for 20 min. Supernatant was utilized for the next centrifugation at 150,000 g and 4 °C for 1 hour (Beckman, Ti 45 rotor). The pellet was resuspended in same cold buffer. As was shown previously^75^, isolated IMs are devoid of OM contamination.

### IM extraction

Cells were were resuspended in a cold buffer (100 mM HEPES, 25 mM MgCl_2_, 1 mM PMSF, pH 7.5). After 2 passages through a French press at 16,000 psi undestroyed cells were removed by centrifugation at 30,000 × g and 4°C for 20 min (RC6 centrifuge, SS-34 rotor). For total membrane fraction isolation, the supernatant was centrifuged at 200, 000 × g at 4 °C for 1 h (Beckman Ti 50.2 rotor). The pellet was resuspended in 10 mM HEPES, 25 mM MgCl_2_, 1 mM PMSF, pH 7.5 and stored at −20 °C.

### Sample Preparation for Blue Native-Electrophoresis (BN-PAGE)

To solubilize membrane protein complexes for BN-PAGE, IM fraction obtained as described above was resuspended by tip sonication in commercial Bio-Rad 4X sample buffer (final 1X contained 50 mM NaCl, 50 mM imidazole/HCl, 2 mM 6-aminohexanoic acid, 1 mM EDTA, pH 7.0) supplemented with 1  mM PMSF, 0.08% glycerol, 2% digitonin or 1% (n-dodecyl β-D-maltoside)(DDM) and 8 mM TrisHCl. The samples were incubated on a rocking platform at 4 °C for 2 h follow by centrifugation at 20,000 × g and 4 °C for 15 min. Supernatant containing solubilized membranes was transferred to fresh tubes and resuspended in 4X sample buffer. After 15 min incubation at 37 °C on a shaker, 20 μl of the probe was used for loading to 4–15% BN-PAGE gel.

### Sample Preparation for SDS-PAGE

For SDS-PAGE solubilized IMs were resuspension in Bio-Rad Laemmli sample buffer with alternating steps of vortexing, incubation at 37 °C and vortexing for 15 min each. To remove non-solubilized components, the samples were centrifuged at 14,000 g at room temperature. SDS-PAGE was carried out by using 3–12% Bio-Rad or Invitrogen gels. Broadening of LacY and OmpF bands is due to presence of multiple folding intermediates caused by incomplete denaturation of membrane proteins. This is a common problem for LacY, OmpF and other membrane proteins and this “fuzziness” could be enhanced by lipid solvation and using semi-native SDS-PAGE. Although higher molecular weight aggregates of OmpF trimers (products of non-productive interactions) can be dissociated by adding 0.5 M NaCl and 5 mM EDTA to the solubilization and sample buffers, these reagents were intentionally omitted in our assays, since the high ionic strength and removal of chelated divalent cations would affect the lipid-protein and protein mono- and hetero-oligomeric interactions crucial for preservation of the trimeric physiologically relevant structure of porin OmpF.

### Immunoblotting

After BN-PAGE or SDS-PAGE, samples were electroblotted onto nitrocellulose membranes and immunosttained with polyclonal antibodies directed against directed against SecY, SecE, SecG, SecA and YidC. Detection was performed with HRP-conjugated goat anti-rabbit antibodies as secondary antibodies and Fluor-S MaxTM MultiImager (Bio-Rad) equipped with a CCD camera and a Nikon 50 mm 1:1.4 AD (F 1.4) lens at the ultrasensitive chemiluminescence setting.

### Enzymatic assays

The enzymatic activity of *E. coli* alkaline phosphatase was measured by colorimetric assay with *p*-nitrophenyl phosphate (pNPP) as a substrate. Pho regulon was induced in LB medium lacking orthophosphate. Phosphate lacking LB media was prepared by resuspension of LB (10 g tryptone, 5 g yeast extract and 10 g NaCl) in 200 ml of distilled water with the addition of 1.8 ml of concentrated NH_4_OH and 28.25 ml of 1 M MgCl_2_. Solution was incubated on rocking platform at 4 °C for 2 h, then media was filtered, pH was adjusted to 7.5, volume was adjusted to 1 liter, and resulting media was autoclaved. The cells were initially grown at 37 °C and 250 rpm in regular LB until mid-log phase (A_600_ = 0.6), centrifuged and then transferred to phosphate lacking media. After 2 washes in this media, cells were left to grow under similar conditions (37 °C, 250 rpm). Aliquots were taken after 15, 30, 45, 60 minutes. Cells then were resuspended in 200 μl of 0.5% thiomersal in 100 mM Tris-HCl with 10 mM MgCl_2_, pH 8.5 and incubated on ice for 5 min to stop all metabolic processes. 5 μl of toluene was added followed by vortexing for 15 s with subsequent addition of 0.5 ml of pNPP in same buffer. Samples were incubated at 37 °C for 30–60 min and the reaction stopped by addition of 2 ml of 0.4 M NaOH. Samples were centrifuged at 20,800 × g and 4 °C for 10 min. Absorption was measured at 400 nm and specific enzymatic activity was calculated.

### Statistical analysis

The results of quantitative experiments are shown as means for independent experiments performed multiple times as indicated. Values are means (± S.D.) from three experiments, where each determination was done in duplicate. Variation between duplicates was ±3%. Images represent three independent experiments.

## Supplementary information

Supplementary Information.
