# Supplementary material for: Cardiolipin is required in vivo for the stability of bacterial translocon and optimal membrane protein translocation and insertion
Source: Sci Rep. 2020 Apr 14;10:6296. doi: 10.1038/s41598-020-63280-5 (PMC7156725; doi:10.1038/s41598-020-63280-5)

**Cardiolipin is required *in vivo* for the stability of bacterial translocon and optimal membrane protein translocation and insertion**

Sergey Ryabichko^1,2*^, Vilena de Melo Ferreira^1,3*^, Heidi Vitrac^1^, Ramziya Kiyamova^4^, William Dowhan^1^ and Mikhail Bogdanov^1,4**^

^1^Department of Biochemistry and Molecular Biology McGovern Medical School at the University of Texas Health Science Center, Houston, Texas 77030, USA

^2^Lewis-Sigler Institute for Integrative Genomics, Princeton University, Princeton, NJ 08540, USA

^3^Monash University, the Monash Institute of Pharmaceutical Sciences

381 Royal Parade, Parkville VIC 3052, Australia

^4^Department of Biochemistry, Biotechnology and Pharmacology, Kazan (Volga Region) Federal University, Institute of Fundamental Medicine and Biology, Kazan, Russian Federation 420008

*Equal contribution

**To whom correspondence should be addressed: Mikhail Bogdanov, Ph.D.

Department of Biochemistry and Molecular Biology, University of Texas Health Science Center, McGovern Medical School, 6431 Fannin St., Suite 6.204, Houston, TX 77030

USA e-mail [mikhail.v.bogdanov@uth.tmc.edu](mailto:mikhail.v.bogdanov@uth.tmc.edu) Tel. 713-500-6123

ORCID <https://orcid.org/0000-0002-7176-8127>

**Uncropped images**


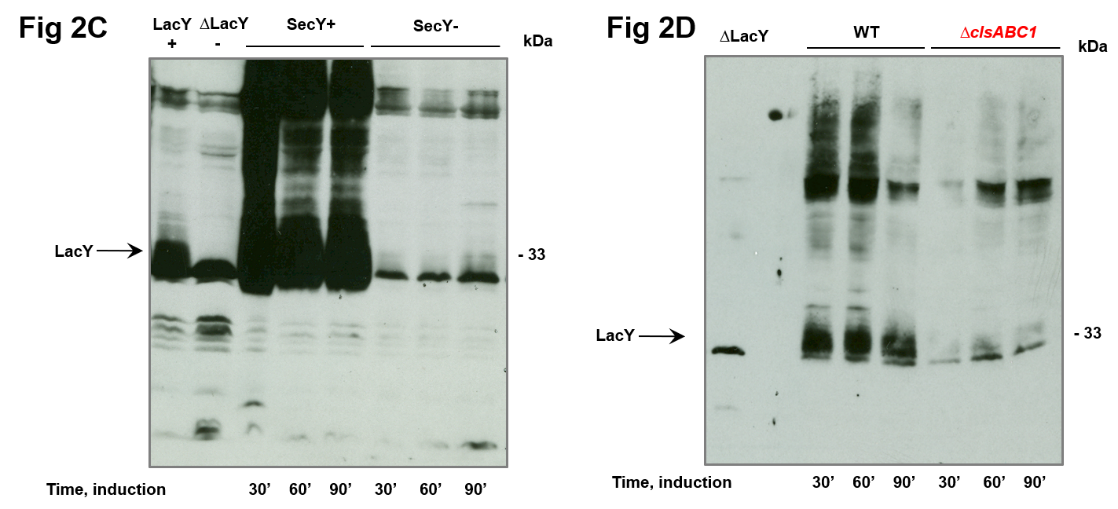


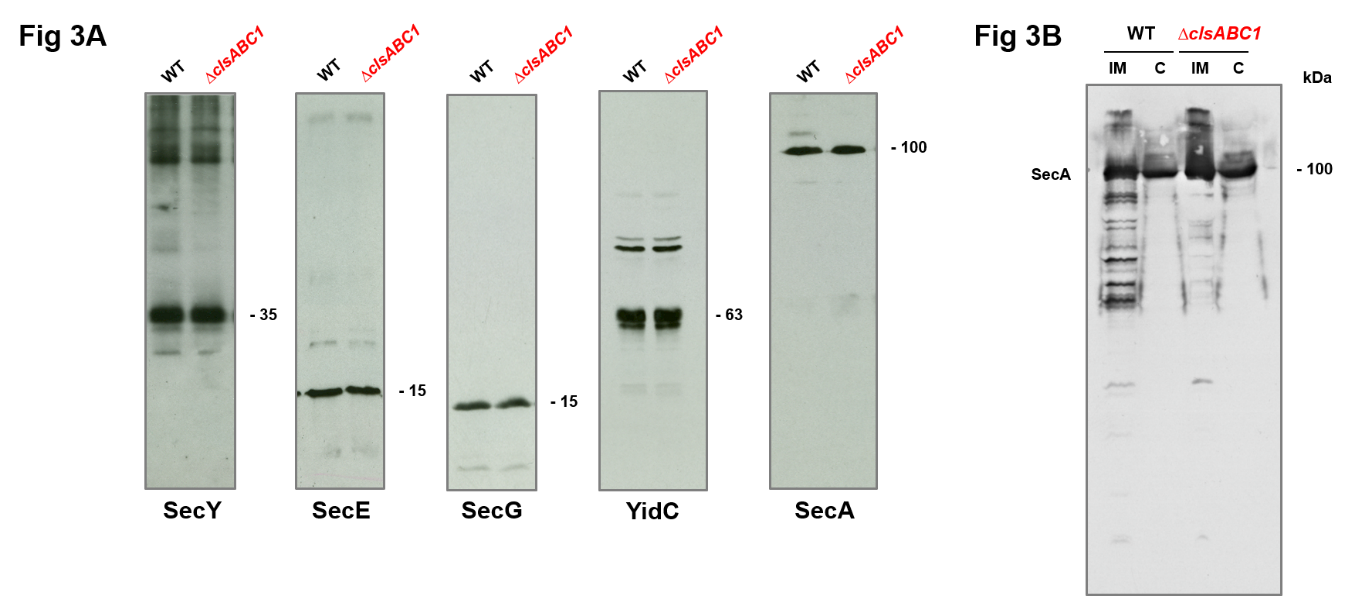


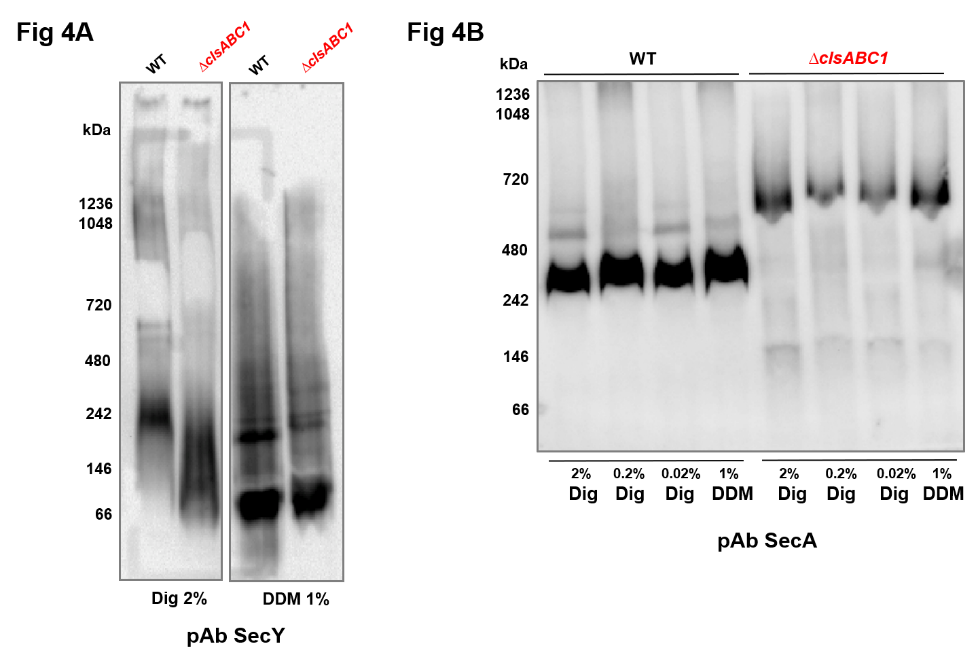

Supplement: Supplementary file 1 — Supplementary Information. [file 41598_2020_63280_MOESM1_ESM.docx]
